# Staufen targets *coracle* mRNA to *Drosophila* neuromuscular junctions and regulates GluRIIA synaptic accumulation and bouton number

**DOI:** 10.1016/j.ydbio.2014.06.007

**Published:** 2014-08-15

**Authors:** Alejandra Gardiol, Daniel St Johnston

**Affiliations:** The WellcomeCRUK Gurdon Institute, University of Cambridge, Tennis Court Road, Cambridge CB2 1QN, United Kingdom

**Keywords:** mRNA localisation, Glutamate receptors, Subsynaptic translation, Neuromuscular junction, Staufen

## Abstract

The post-synaptic translation of localised mRNAs has been postulated to underlie several forms of plasticity at vertebrate synapses, but the mechanisms that target mRNAs to these postsynaptic sites are not well understood. Here we show that the evolutionary conserved dsRNA binding protein, Staufen, localises to the postsynaptic side of the *Drosophila* neuromuscular junction (NMJ), where it is required for the localisation of *coracle* mRNA and protein. Staufen plays a well-characterised role in the localisation of *oskar* mRNA to the oocyte posterior, where Staufen dsRNA-binding domain 5 is specifically required for its translation. Removal of Staufen dsRNA-binding domain 5, disrupts the postsynaptic accumulation of Coracle protein without affecting the localisation of *cora* mRNA, suggesting that Staufen similarly regulates Coracle translation. Tropomyosin II, which functions with Staufen in *oskar* mRNA localisation, is also required for *coracle* mRNA localisation, suggesting that similar mechanisms target mRNAs to the NMJ and the oocyte posterior. Coracle, the orthologue of vertebrate band 4.1, functions in the anchoring of the glutamate receptor IIA subunit (GluRIIA) at the synapse. Consistent with this, *staufen* mutant larvae show reduced accumulation of GluRIIA at synapses. The NMJs of *staufen* mutant larvae have also a reduced number of synaptic boutons. Altogether, this suggests that this novel Staufen-dependent mRNA localisation and local translation pathway may play a role in the developmentally regulated growth of the NMJ.

## Highlights

• Stau mutant neuromuscular junctions have reduced GluRIIA levels and fewer boutons.
• Stau regulates cora mRNA localisation and translation.
• Cora and osk mRNA share localisation factors and may localise by similar mechanisms.

## Introduction

mRNA localisation is a widespread mechanism for targeting proteins to a specific region within a cell, and can be coupled to translational regulation to allow the local control of gene expression (St Johnston, 2005; Holt and Bullock, 2009). This mechanism has been proposed to play an important role in the nervous system, where the translation of dendritically localised mRNAs near synapses is thought to contribute to activity-dependent synaptic remodelling during long-term potentiation or depression. It has been known for many years that polyribosomes are present in dendrites in the vicinity of synapses. In addition, more than 20 mRNAs have been found to be dendritically localised, most of which encode proteins that regulate synaptic structure or function, consistent with the idea that their local translation modifies synaptic strength (Sutton and Schuman, 2006; Zukin et al., 2009; Doyle and Kiebler, 2011; Kindler and Kreienkamp, 2012). This has been most clearly demonstrated in the case of CaMKIIα, where a mutant RNA lacking dendritic targeting signals leads to a reduction of protein levels in distal dendrites and impaired long term potentiation and memory (Miller et al., 2002).

Little is known about the mechanisms that direct the postsynaptic localisation of mRNAs in neurons, but live imaging of CaMKIIα and Arc mRNAs has revealed that they undergo rapid bidirectional movements suggestive of motor-dependent transport along microtubules (Rook et al., 2000; Dynes and Steward, 2007). In support of this view, a number of dendritic mRNAs are found in RNP particles that co-purify with the plus end-directed microtubule motor protein, Kif5 (Kanai et al., 2004). mRNAs are usually targeted to dendrites by localisation elements in their 3′UTRs, which must be recognised by RNA binding proteins (RBPs) that link them to the transport machinery and regulate translation (Kindler and Kreienkamp, 2012).

Amongst the proteins that are suspected to play a direct role in dendritic mRNA transport are the vertebrate Staufen proteins, which contain multiple copies of a conserved dsRNA-binding domain (dsRBD) (St Johnston et al., 1992; Kiebler et al., 1999; Marion et al., 1999; Wickham et al., 1999). In cultured neurons, Staufen forms ribonucleoprotein particles that are transported along microtubules into dendrites, whereas dominant negative versions of the protein remain in the soma and reduce levels of RNA and ribosomes in dendrites (Kiebler et al., 1999; Köhrmann et al., 1999; Tang et al., 2001; Kim and Kim, 2006). Moreover, many dendritically-localised mRNAs co-immunoprecipitate with embryonic rat brain Staufen (Heraud-Farlow et al., 2013). Suppressing the expression of either of the two rodent Staufen orthologues affects the morphology of dendritic spines, which are abnormally shaped and immature (Goetze et al., 2006; Vessey et al., 2008). Although *stau 1* mutant mice show no obvious behavioural deficits, RNAi-mediated knock down of Stau1 function in hippocampal slices impairs long term potentiation, whereas knockdown of the second Stau gene disrupts long term depression (Lebeau et al., 2008, 2011; Vessey et al., 2008)

Most of our understanding of the role of Staufen in mRNA localisation comes from *Drosophila*, where it participates three distinct mRNA localisation pathways. First, Staufen forms a complex with *oskar* mRNA and is essential for the kinesin-dependent transport of the mRNA to the posterior of the oocyte and for its local translation at the posterior cortex (Ephrussi et al., 1991; Kim-Ha et al., 1991, 1995). Mutants that disrupt Staufen RNA-binding strongly reduce the localisation of *oskar* mRNA, whereas deletion of the fifth dsRBD, which has the conserved structure of the dsRBD but does not bind dsRNA, prevents the translation of *oskar* mRNA once it has localised (Rongo et al., 1995; Micklem et al., 2000). Second, Staufen is recruited to the *bicoid* 3′UTR by the ESCRT-II complex, and is required for the anchoring of the mRNA at the anterior of the oocyte during late oogenesis (St Johnston et al., 1989; Ferrandon et al., 1994; Weil et al., 2006; Irion and St Johnston, 2007). In addition to its role localising *bicoid* and *oskar* at opposite poles, Staufen is also required for the actin-dependent localisation of *prospero* mRNA to the basal side of asymmetrically dividing neuroblasts (Li et al., 1997; Broadus et al., 1998). This depends on the binding of the fifth dsRBD of Staufen to Miranda, which targets *prospero* RNA/Staufen complexes to the basal cortex (Fuerstenberg et al., 1998; Matsuzaki et al., 1998; Schuldt et al., 1998; Shen et al., 1998).

Given its well-characterised role in mRNA localisation*,* we set out to investigate whether *Drosophila* Staufen plays a role in the targeting of mRNAs to synapses using the neuromuscular junction (NMJ) as a model. Although the post-synaptic cell is a muscle, the NMJ has the advantage of being a well-characterised glutamatergic synapse that displays developmental and activity-dependent synaptic plasticity, and shares some aspects of its cell biology and physiology with vertebrate central nervous system excitatory synapses (Schuster, 2006).

## Results

### Staufen is localised to the postsynaptic compartment of the NMJ

**Fig. 1:**
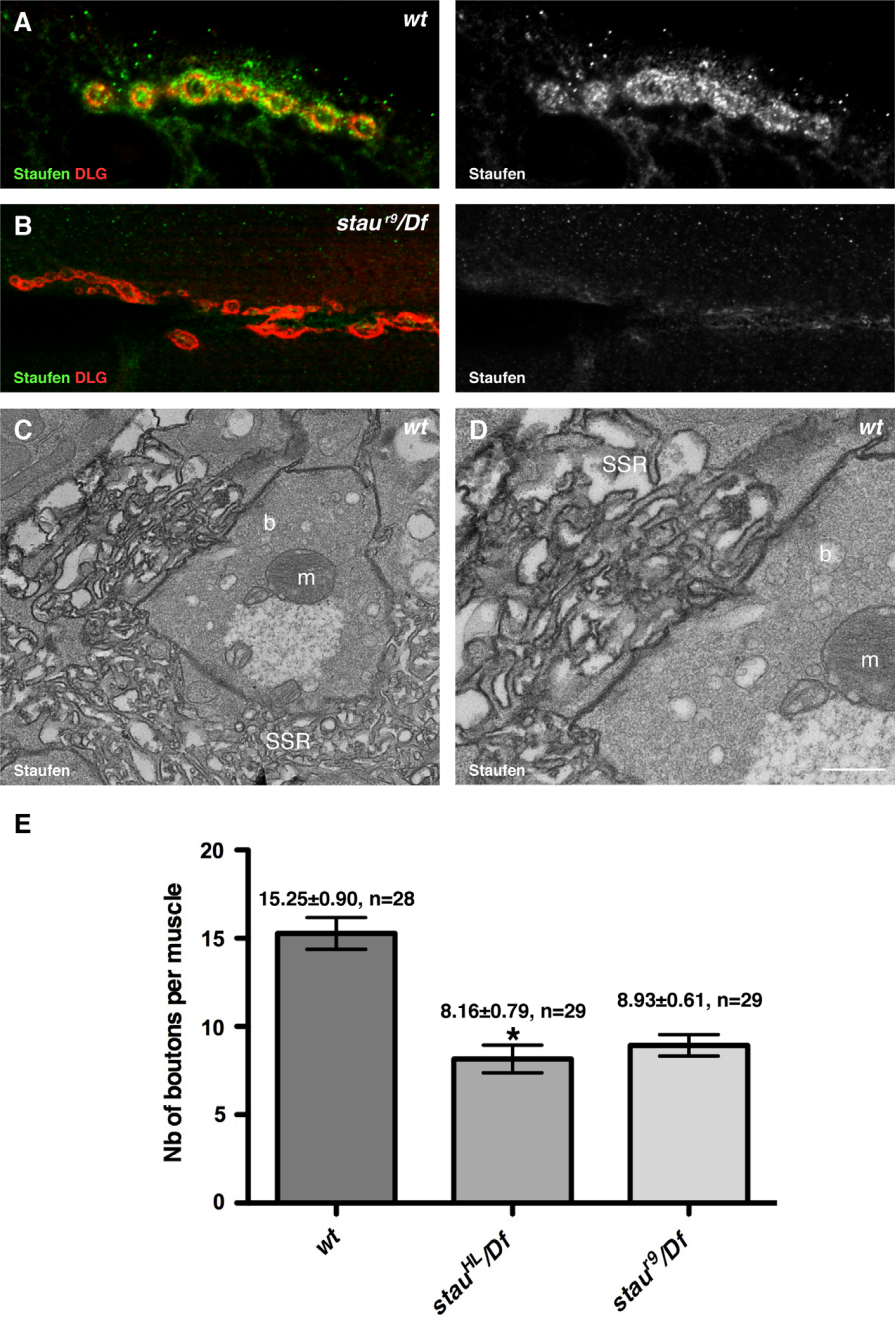
Loss of postsynaptic Staufen reduces bouton numbers. (A and B) Confocal images of boutons labelled for DLG (red) and Staufen (green). Staufen localises around the DLG-positive boutons on the postsynaptic side of the NMJ of a wild type (wt) muscle (muscle 4), but is absent in a null mutant (*stau*^*r9*^/*Df*), here an example of the NMJ between muscles 6/7. (C and D) Electron micrograph of a longitudinal section through part of the NMJ of a wild type muscle 6. (C) The DAB precipitate associated with Staufen immunoreactivity is restricted to the sub-synaptic reticulum (SSR) surrounding a synaptic bouton (b). (D) Enlargement of a region of (C) showing the strong Staufen signal around the SSR, but not in the presynaptic bouton (b). Note the presence of a mitochondria (m) and the abundance of round, clear synaptic vesicles within boutons. (E) Quantification of Ib bouton number in the NMJs between muscles 6/7 in wild type, *stau*^*HL*^/*Df* and *stau*^*r9*^/*Df* (^⁎^ indicates *p*<0.0001). Scale bar (A: 7 µm; B: 25 µm; C: 0.7 µm; D: 0.4 µm).

In the third instar larva, each muscle is a single multinucleated cell that is simultaneously innervated by up to four motorneurons that form synapses en passant after defasciculating from the motor nerve. The NMJ is considered to be the assembly of regularly spaced swellings called boutons that are formed by the axons. Each presynaptic bouton contains in average 20–40 active zones where synaptic vesicles are docked, which are faced by a postsynaptic differentiation (PSD) where neurotransmitter receptors cluster forming junctional excitatory synapses (Budnik, 1996; Schuster, 2006; Thomas and Sigrist, 2012). In double immunofluorescent stainings in third instar larva fillets, an antibody against Staufen labelled the periphery of type I boutons, outside the staining for Discs Large (Dlg), a MAGUK protein belonging to the PSD-95, Sap90/97 family that decorates the subsynaptic reticulum (Lahey et al., 1994; Guan et al., 1996) (SSR, Fig. 1A). The Staufen staining was specific, as it was absent from the NMJs of *staufen* null mutant larvae (Fig. 1B). We also performed pre-embedding immune-EM using HRP-conjugated anti-Staufen antibodies and diaminobenzidine (DAB) staining, which precipitates on membranes when oxidised. The electron-dense DAB signal was found around the invaginations of the muscle membrane that form the SSR beneath glutamatergic type I boutons, whereas no signal could be detected in the presynaptic element (Fig. 1C and D). Staufen therefore localises on the postsynaptic side of all type I boutons in third instar larval NMJs.

### Staufen mutants have a reduced number of boutons

During larval development, the muscle size increases. In order to maintain efficient innervation, the NMJ expands accordingly and more boutons are added (Budnik, 1996). The NMJs of Staufen mutant larvae appear less developed than their wild type counterparts, and we therefore quantified the number of boutons per NMJ in different *staufen* allelic combinations. The *staufen*^*r9*^ allele and a deficiency (*Df*) have molecular lesions that entirely abolish *staufen* expression, whereas *staufen*^*HL*^ produces a truncated form of Staufen missing the fifth double-stranded RNA binding domain (see Section 4). In wild type larvae, the NMJ established between muscles 6/7 had as an average of 15 type Ib boutons (Fig. 1E; *wt*: 15.25±0.90, *n*=28). In *staufen* mutant larvae, there was almost a 50% reduction in the number of boutons (Fig. 1E; *HL/Df*: 8.16±0.79, *n*=29; *r9/Df*:, 8.93±0.61, *n*=29). Mutants that fail to incorporate new boutons during the development have deformed NMJs with poorly defined boutons, as if they have been mechanically stretched (Zito et al., 1999). The NMJs in *staufen* mutants have poorly defined boutons of this type with long linear stretches, indicating that Staufen is involved in the process that increases bouton number during the development of the NMJ.

### Staufen regulates GluRIIA and GluRIIB levels at the NMJ

**Fig. 2:**
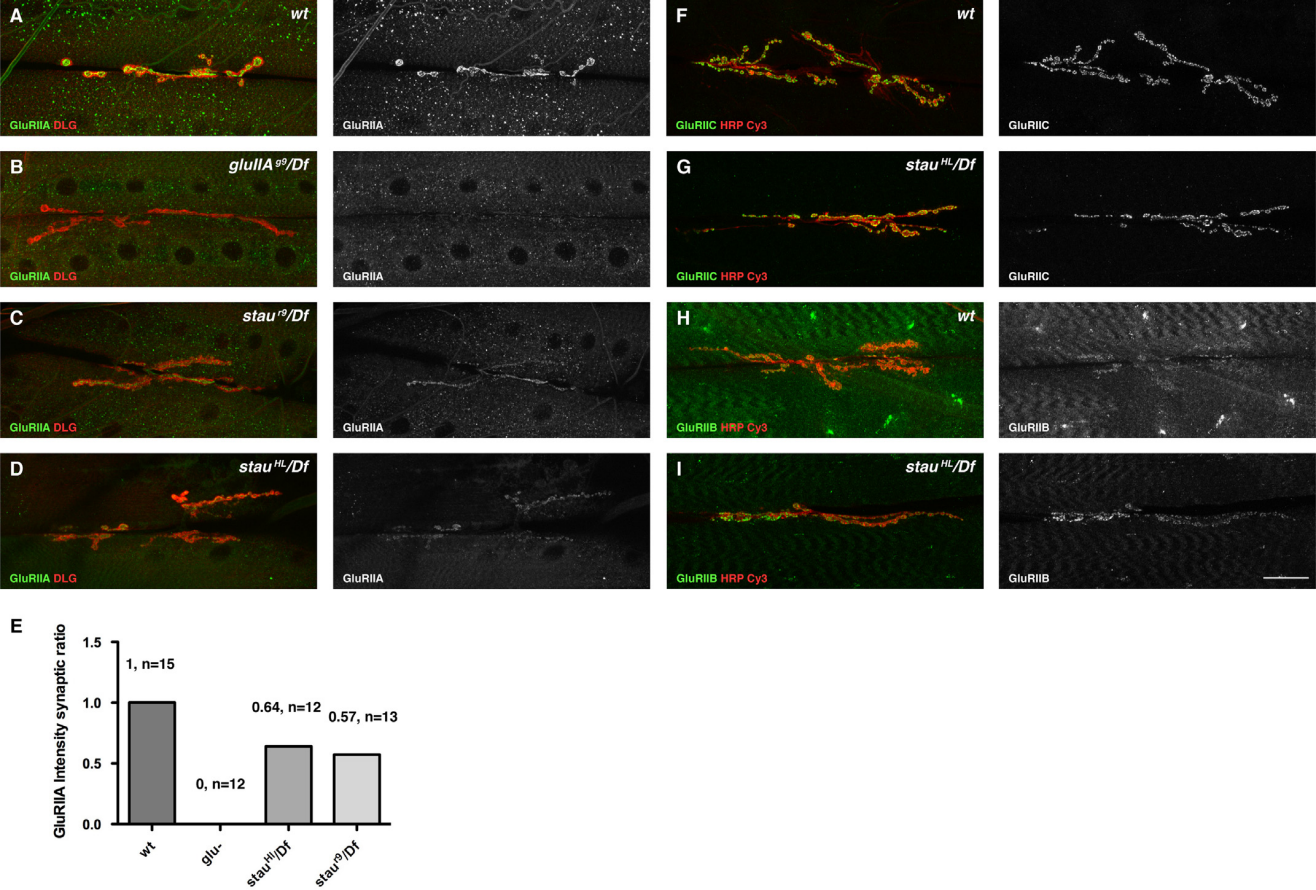
*stau* mutants cause a decrease in GluRIIA levels. (A–D) Synaptic boutons labelled for DLG (red) and GluRIIA (green) at NMJs between muscles 6/7. (A) Wild type; (B) *gluIIA/Df*; (C) *stau*^*r9*^/*Df*; (D) *stau*^*HL*^/*Df*. (E) Quantification of the ratio of the mean intensities of the synaptic vs. extra-synaptic GluRIIA signals. *stau* mutant NMJs have reduced levels of GluRIIA compared to wild type (wt), and GluRIIA mutant boutons had no detectable signal (Glu-). (F–I) Localisation of GluRIIC (F and G, green) and GluRIIB (H and I, green) subunits at the NMJs between muscles 6/7, labelled with Cy3 anti-HRP (red). (F) GluRIIC forms postsynaptic clusters in wild type larvae. (G) The GluRIIC clusters have a similar abundance and distribution in *stau*^*HL*^/*Df*. (H) GluRIIB is weakly localised in clusters at the NMJ (red) in wild type. (I) GluRIIB clusters at the NMJs seem brighter and more abundant in *stau*^*HL*^/*Df* mutants. Scale bar (A–D: 37 µm; F–I: 24 µm).

The NMJ contains two types of GluRs similar in sequence to vertebrate AMPA and Kainate receptors, GluRIIA and GluRIIB, each of which is a hetero-tetramer of three common subunits, GluRIIC, IID, IIE, and either a GluRIIA or a GluRIIB subunit (Schuster et al., 1991; Chang et al., 1994; Petersen et al., 1997; Marrus and Diantonio, 2004; Featherstone et al., 2005; Qin et al., 2005). It has been reported that in some cases, altered levels of GluRIIA can be related to a reduction in the number of synaptic boutons (Sigrist et al., 2000, 2002, 2003). We therefore investigated by immunocytochemistry whether GluR abundance at the NMJ was affected. A polyclonal antibody directed against the N-terminal region of GluRIIA detected discreet clusters on the postsynaptic side of the NMJ (Saitoe et al., 1997) (Fig. 2A). The antibody, as described previously, was specific for the GluRIIA subunit since no synaptic clusters could be detected in *glurIIa* null mutants (Fig. 2B). The intensity of the synaptic GluRIIA signal was greatly reduced in *staufen*^*r9*^*/Df* and *staufen*^*HL*^*/Df* larvae (Fig. 2C–E).

In wild type NMJs, the GluRIIA and GluRIIB subunits compete for their association with the GluRIIC subunit and for their subsequent incorporation into the NMJ, and mutants lacking either GluRIIA or GluRIIB are therefore viable, whereas simultaneous deletion of both genes results in lethality (Petersen et al., 1997; DiAntonio et al., 1999). In contrast, elimination of either GluRIIC, D or E subunits leads to embryonic lethality (Marrus et al., 2004; Featherstone et al., 2005; Qin et al., 2005). Since *staufen* mutant larvae are viable and motile, GluRIIB presumably compensates for the reduction in GluRIIA by associating with the structural subunits (GluRIIC,D or IIE). Indeed, *staufen* mutants showed normal levels of GluRIIC (Fig. 2F and G). GluRIIB synaptic clusters were not affected in *staufen* mutants (Fig. 2H and I). This suggests that, as described before, GluRIIB clusters could have compensated for the reduction in GluRIIA. Our immunostainings indeed suggest that GluRIIB levels are increased. Thus, Staufen modulates the relative abundance of the two GluRs at the NMJ, raising the question of the mechanisms that underlie this regulation.

### Staufen does not regulate the translation of GluRIIA mRNA

**Fig. 3:**
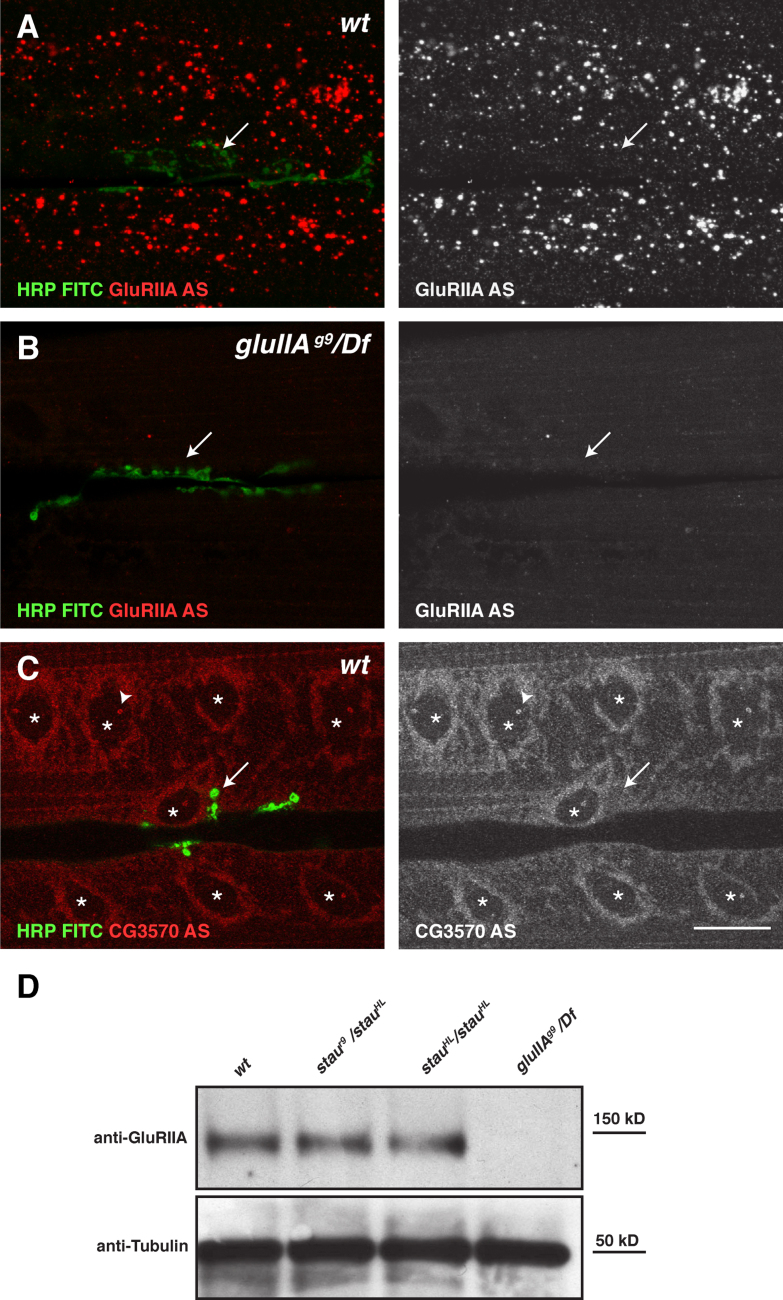
Staufen does not regulate the localisation or translation of *gluRIIA* mRNA. (A–C) in situ hybridizations (red) in muscles 6/7 with NMJs labelled by FITC anti-HRP (green). (A) *gluRIIA* mRNA localises to cytoplasmic puncta in muscles 6 and 7 and is not associated with the NMJ (arrow, green). (B) The *gluRIIA* mRNA signal is specific, since it is absent in the mRNA null mutant, *gluIIA*^g9^/*Df*. (C) CG3570 mRNA localises around nuclei (asterisks) but not to the NMJ (arrow, green). Single dots in the nuclei may correspond to sites of transcription (arrowheads). (D) Western blot of larval fillets probed for GluRIIA and alpha-Tubulin as a loading control. The GluRIIA antibody detects a 150 Kd band of equivalent intensity in wild type (wt), *stau*^*HL*^/ *stau*^*HL*^ and *stau*^r9^/ *stau*^*HL*^ that is absent in the *gluRIIA* null mutant (*gluIIA*^*g9*^*/Df*). Scale bar (A–C: 25 µ).

Since the only conserved domains of Staufen are dsRNA-binding domains and all of its known functions in *Drosophila* involve mRNA localisation and/or local translation, it presumably controls glutamate receptor levels at the NMJ by regulating an mRNA (Micklem et al., 2000). A strong candidate for a target of Staufen in the muscle is *gluRIIA* mRNA, since this transcript has been reported to localise to the subsynaptic compartment of the NMJ, where it has been proposed to be translated in response to activity (Sigrist et al., 2000, 2002, 2003). We therefore performed in situ hybridisations (ISH) for *gluRIIA* mRNA using the previously published probes and methods, but did not observe any specific enrichment at the NMJ. Indeed, we used an alkaline phosphatase based ISH method using a fluorescent substrate and detected GluRIIA mRNA as speckles distributed within the cytoplasm of larval muscles (Fig. 3A). This signal was absent in *gluRIIA* mRNA null mutants, confirming its specificity (Fig. 3B). In order to test our ISH method, we also performed an ISH against *CG3570* mRNA, which shows a perinuclear localisation in the ovary, and observed a similar perinuclear localisation in the muscle syncytium, confirming the reliability of this technique (Fig. 3C). Thus, *gluRIIA* mRNA does not localise to the NMJ and is found instead in cytoplasmic puncta in agreement with other reports (Currie et al., 1995; Karr et al., 2009; Ganesan et al., 2011). GluRIIA is a transmembrane protein and must be synthesised in the endoplasmic reticulum (ER) before oligomerising with the other subunits during its journey along the secretory pathway through the Golgi apparatus to the plasma membrane (Barry and Ziff, 2002). The ER is mainly localised around the nuclei of the muscle and is not detectable in the vicinity of the NMJ, whereas the dispersed Golgi ministacks are uniformly distributed in the cytoplasm (Figure S1A–D). The absence of the secretory apparatus around the NMJ makes it unlikely that secreted proteins such as GluRIIA could be locally translated and secreted at the synapse (Gardiol et al., 1999; Hanus and Ehlers, 2008).

Since GluRIIA protein staining is strongly reduced in *staufen* mutant NMJs, we next examined whether Staufen plays a role in GluRIIA translation. Western blots on wild type and *staufen* mutant fillets using the polyclonal GluRIIA antibody revealed that GluRIIA protein levels in *staufen* mutants are similar to those in wild type (Fig. 3D). Thus, Staufen does not appear to regulate the localisation or translation of *gluRIIA* mRNA or the stability of GluRIIA protein. The reduction in junctional GluRIIA clusters in *staufen* mutants must therefore be due to a defect in the trafficking or anchoring of the receptor.

### Staufen regulates the synaptic localisation of Coracle

Since Staufen does not appear to play a direct role in GluRIIA localisation, we examined whether *staufen* mutants affect any molecules known to play a role in the clustering of GluRIIA at the NMJ (Parnas et al., 2001; Albin and Davis, 2004; Menon et al., 2004; Chen et al., 2005; Pielage et al., 2006). However, Pak, Pumilio and Spectrin levels at the NMJ are unchanged in *staufen* mutant NMJs (7data not shown).

**Fig. 4:**
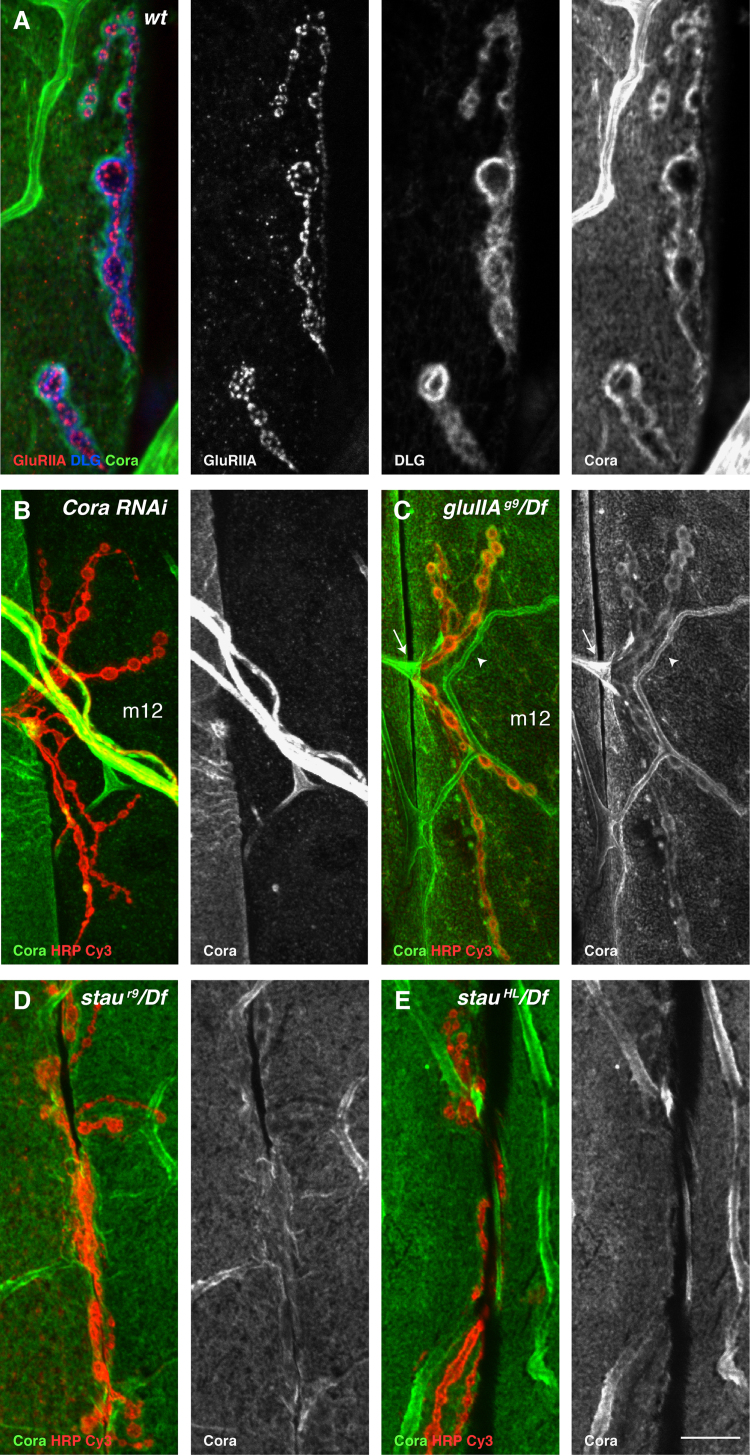
Coracle localisation around the NMJ is reduced in *stau* mutants. (A–E) Confocal images of synaptic boutons labelled for DLG (blue), GluRIIA (red) and Coracle (green). (A) Coracle localises around the periphery of the NMJ just outside the ring of DLG that surrounds the GluRIIA clusters (B) Expression of *cora*-RNAi in muscle 12 (m12) abolishes the Coracle signal around the NMJ and in the cytoplasm of this muscle. (C) Coracle localises normally around the boutons in a *gluRIIA* null mutant. Note the strong Coracle signal in muscle 12 (m12). Coracle antibody strongly stains trachea (arrowhead) and glia (arrow). (D–E) Coracle localisation around the NMJ is strongly reduced in *stau*^*r9*^/*Df* (D) and *stau*^*HL*^/*Df* (E). Scale bar (A: 8 µ; B, C: 12 µm; D, E: 15 µm).

Coracle (the *Drosophila* orthologue of vertebrate 4.1 protein) is required for GluRIIA clustering in the embryo and interacts directly with the C-terminus of GluRIIA (Chen et al., 2005). Coracle is strongly expressed in the glia and trachea (Fig. 4A) (Fehon et al., 1994; Bogdanik et al., 2008). In addition, a polyclonal antibody against Coracle reveals a rim of staining around the boutons on the postsynaptic side of the NMJ (Fig. 4A). To test the specificity of the postsynaptic staining, we knocked down Coracle expression in the muscles using the Gal4/UAS system (Brand and Perrimon, 1993). Gal4 lines that drive expression in all muscles (24B-Gal4, Mhc82-Gal4, BG57-Gal4) are lethal in combination with UAS-*cora* RNAi, but animals expressing *cora* RNAi under the control of the muscle 12 specific driver, *5053A-Gal4* (Ritzenthaler et al., 2000) are viable until adulthood. Coracle staining was lost from muscle 12 of all *5053A-Gal4, UAS-cora RNAi* third instar larvae, including the staining around the boutons, whereas the glia and tracheal stainings were unaffected, confirming that the postsynaptic labelling is specific (Fig. 4B).

The localisation of Coracle at the NMJ of the third instar larva is independent of GluRIIA, since it localises normally in *gluRIIA* null mutants (Fig. 4C). By contrast, the amount of Coracle around the NMJ is greatly reduced in *staufen*^*r9*^*/Df* and *staufen*^*HL*^*/Df* mutant larvae (Fig. 4D and E). Thus, Staufen plays a role in the localisation of Coracle around the boutons, which may account for the reduction in GluRIIA levels at the NMJ in *staufen* mutants.

### Coracle mRNA localises to the NMJ

**Fig. 5:**
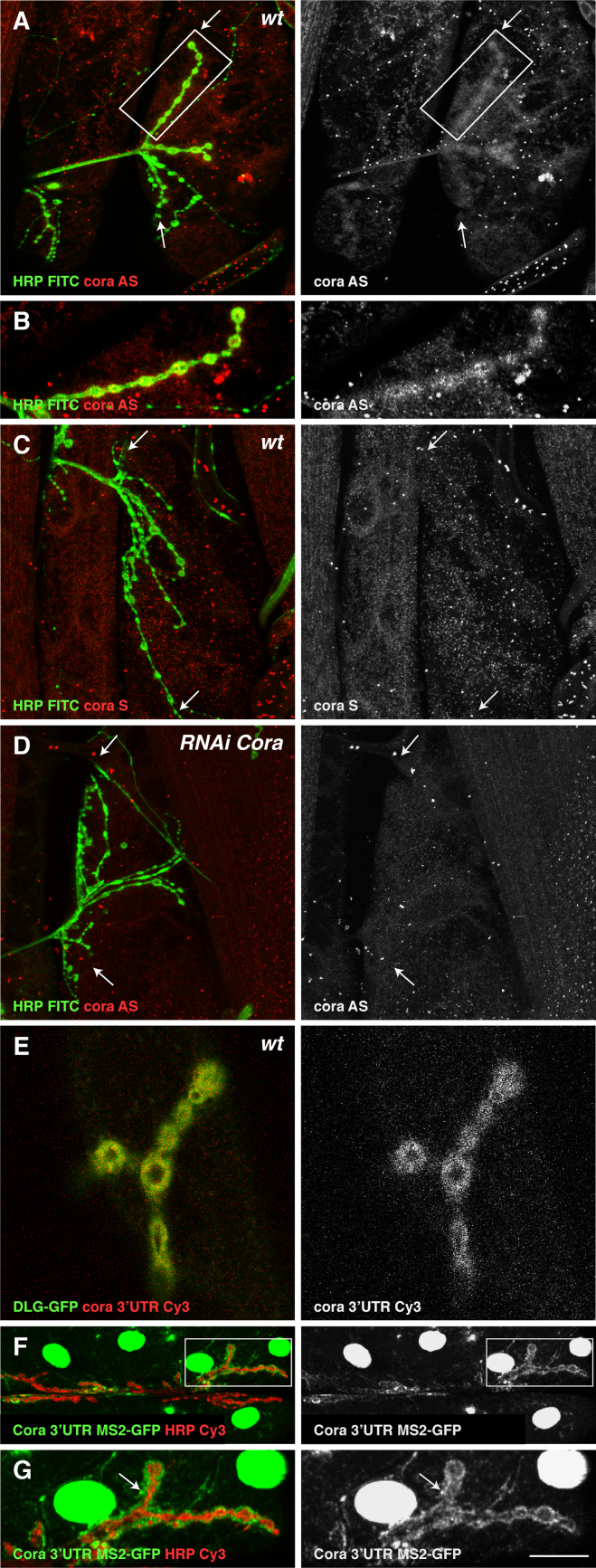
*cora* mRNA localises to the NMJ. (A) Muscle 12 after in situ hybridisation (ISH) with a *cora* anti-sense probe (red). *cora* mRNA localises around the NMJ (between arrows) labelled with FITC anti-HRP (green). (B) Higher magnification of boxed region in (A) showing the particulate postsynaptic ISH signal. (C) ISH with a control *cora* sense probe (red). (D) Expression of *cora*-RNAi in muscle 12 (m12) abolishes the specific *cora* mRNA signal around the NMJ (between arrows). (E) Fixed fillets 2 hs after micro-injection of *cora* 3׳UTR-Cy3 into a live DLG-GFP expressing muscle. *cora* 3׳UTR-Cy3 (red) can be detected postsynaptically at NMJs labelled in green by DLG-GFP. (F) Anti-GFP staining of a muscle expressing *cora* 3׳UTR-10xMS2bs and MS2-GFP (green). The MS2-GFP labelled *cora* RNA localises around the periphery of the boutons, which are labelled with Cy3 anti-HRP (red). (G) Higher magnification view of boxed region in (F) to show the punctate distribution of *cora* mRNA around the NMJ (arrow). Scale bar (A: 24 µm; B: 12 µm; C–E: 24 µm; F: 27 µm; G: 12 µm).

The rim of Coracle around the boutons is reminiscent of the localisation of Staufen protein, raising the possibility that Staufen regulates the localisation of Coracle protein by regulating the localisation and/or the translation of *cora* mRNA. Using a highly sensitive ISH method based on the enzymatic detection of digoxigenin labelled probes with a fluorescent substrate, we asked whether we could detect *coracle* mRNA in the third instar larval muscles. An antisense *coracle* probe gave a diffuse staining throughout the muscle with a significant enrichment around the boutons of the NMJ (Fig. 5A and B). This signal was specific for *cora* mRNA, since it was not detected with *coracle* sense probes and it was abolished in muscle 12 of *UAS-cora RNAi/5053A-Gal4* larvae (Fig. 5C and D).

We used two additional approaches to confirm the localisation of *cora* mRNA at the NMJ. First, we developed an injection assay based on methods used in *Drosophila* embryos, in which fluorescently-labelled mRNAs were injected into muscles of living larvae expressing Dlg::GFP to label the NMJs (Ferrandon et al., 1994; Bullock et al., 2003). After two hours, the fillets were fixed and the injected muscles imaged on a confocal microscope. When we injected the highly conserved *coracle 3′UTR* mRNA, it specifically localised in a rim around the boutons of the NMJ, whereas labelled *gluRIIA 3′UTR* mRNA did not localise to the NMJ after injection (Fig. 5E and data not shown). Secondly, we generated a transgenic line expressing the *cora 3׳UTR* fused to five tandem copies of the MS2-binding site under the control the Gal4/UAS system. This allows the in vivo labelling of the RNA when crossed to flies expressing MS2-GFP, which binds with high affinity to the MS2 binding sites, but is retained in the nucleus by a nuclear localisation signal if not bound to an RNA (Belaya and St Johnston, 2011). The MS2-GFP labelled *cora’ 3׳UTR* showed a very similar localisation to injected *cora 3׳UTR*, forming a rim around the boutons (Fig. 5F and G). By contrast a *gluRIIA 3׳UTR-MS2* fusion mRNA showed no specific localisation in this assay (data not shown). These results confirm that *coracle* mRNA is specifically targeted to the NMJ, and demonstrate that its localisation is directed by elements in its *3*׳UTR, ruling out the possibility that it is localised by a co-translational mechanism.

**Fig. 6:**
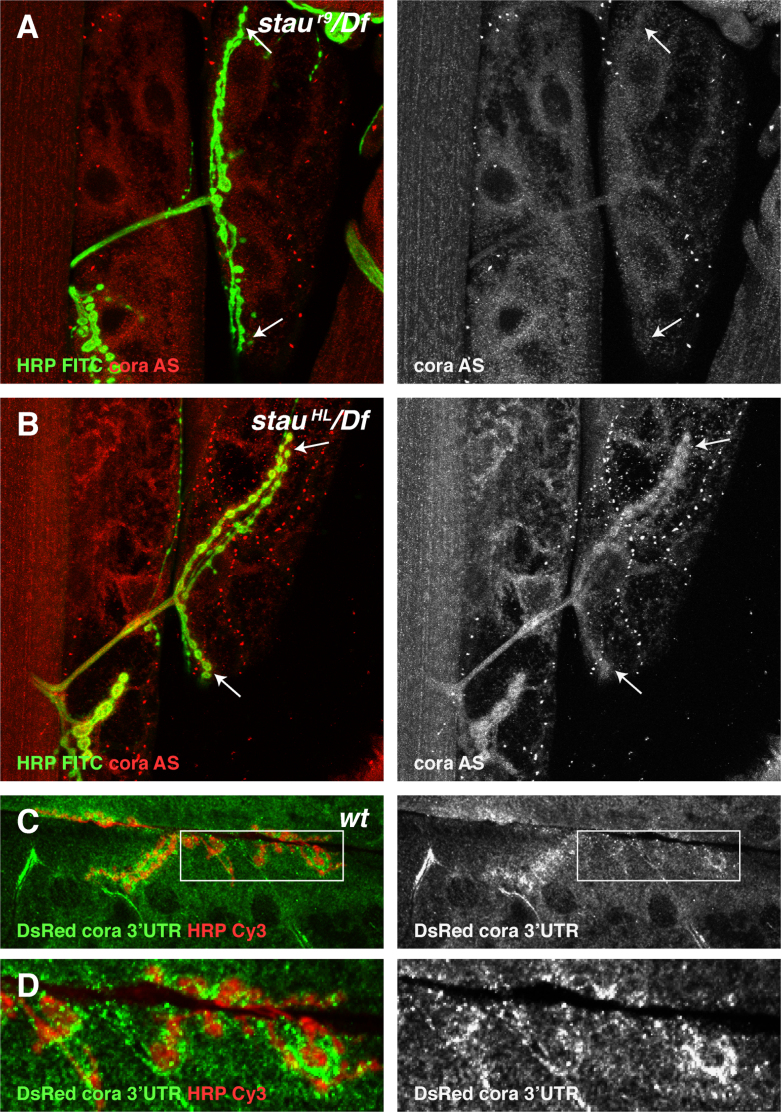
Staufen is required for the localisation and local translation of *cora* mRNA. (A) Muscle from a *stau*^*r9*^/*Df* larva hybridised with a *cora* antisense probe. *cora* mRNA (red) is not localised around the NMJ, labelled with FITC anti-HRP (green, between arrows) in the absence of Staufen protein. (B) *stau*^*HL*^/*Df* hybridised with a *cora* antisense probe. *cora* mRNA localises normally around the NMJ (between arrows) in this mutant, which removes Staufen dsRBD5. (C) Anti-dsReD staining of a wild type larva expressing the translational reporter Timer dsRed-*cora* 3׳UTR. dsRed antibody staining (green) is diffusely localised around the boutons of the NMJ labelled with Cy3 anti-HRP. (D) Higher magnification of boxed region in (C) showing the diffuse postsynaptic dsRED signal (green). Scale bar (A, B: 24 µm; C: 37 µm, D: 15 µm;).

In a *staufen* null mutant combination, *staufen*^*r9*^*/Df, cora* mRNA is no longer localised around the boutons, demonstrating that Staufen is necessary for the targeting of the RNA to the NMJ (Fig. 6A). By contrast, the mRNA localises normally in the hypomorphic mutant, *staufen*^*HL*^*/Df,* which lacks the dsRBD5 domain, despite the strong effect of this mutant on Coracle protein (Fig. 6B). This is reminiscent of the effect of *staufen*^*HL*^ on *oskar* mRNA, where the mRNA is localised normally to the posterior of the oocyte but no Oskar protein is produced (Micklem et al., 2000). Thus, Staufen is required for *cora* mRNA localisation at the NMJ, while the conserved dsRBD5 may be necessary for its translation once the mRNA has been localised.

To test more directly whether *cora* mRNA is locally translated at the NMJ, we generated a translational reporter that is expressed in the muscle under the control of the *cora 5′* and *3′* untranslated regions. Membrane-tethered reporters, such as myristoylated-GFP accumulate on the extensive membrane invaginations of the subsynaptic reticulum, regardless of where they are expressed in the cell (data not shown). We therefore used the age-dependent fluorescent protein, dsRed-E5, which should diffuse only slowly, as it is tetrameric, and has the additional advantage that it has been reported to change its emission from green to red over time, providing a convenient way to measure translation rates (Terskikh et al., 2000). In the environment of the *Drosophila* muscle, however, dsRed-E5 did not change colour with time. Antibody staining for dsRed revealed protein expression at a subset of the boutons of wild type larvae expressing *cora 5׳UTR*-dsRed-E5-*cora 3׳UTR* (Fig. 6C and D). Thus, the untranslated regions of *cora* are sufficient to direct the localisation and local translation of a heterologous protein at the NMJ.

**Fig. 7:**
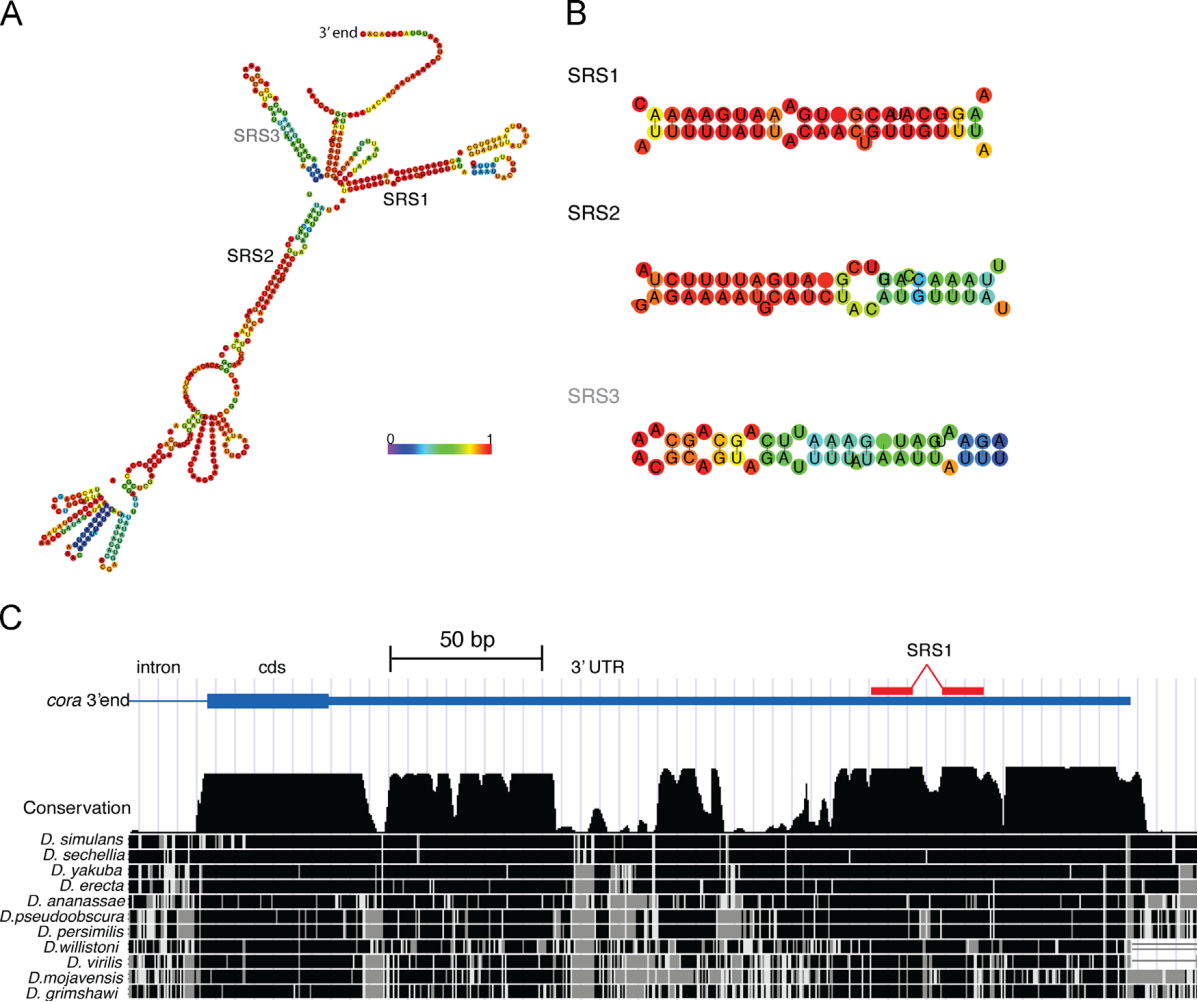
The *cora* 3׳UTR contains two double-stranded regions that match the consensus for Staufen recognised structures. (A) The predicted minimum free energy structure of the *cora* 3׳UTR generated by RNAfold. The RNA used for the folding algorithm also includes the last 50 bases of the coding region to avoid creating an artificial free end at the 5׳end of the 3׳UTR. The colour scale represents the probability that each base is in the state shown in the model (paired or unpaired) calculated by RNAplfold. (B) Two double-stranded regions, SSR1 and SSR2 in the predicted structure match the consensus for Staufen recognised structures, as well as a third region that includes part of the coding region (SRS3, grey). (C) A diagram showing the degree of conservation of the 3׳end of the coracle locus across the *Drosophila* genus using phylogenetic shadowing. The image was downloaded from the UCSC genome browser (*http://genome.ucsc.edu*) and shows the conservation track created by the phastCons package using a grey scale to show the degree of conservation. Large regions of the 3׳UTR are nearly as strongly conserved as the coding region, including the two regions that base pair to form SRS1. Scale bar (C:50nt).

Altogether, it seems likely that Staufen associates directly with the *cora* 3׳UTR. There is no straightforward in vitro assay for Staufen RNA binding, however, as it contains four functional copies of the double-stranded RNA binding domain (dsRBD), each of which binds in a sequence-independent manner to 12 bp of dsRNA, leading to the proposal that its specificity in vivo is conferred by the simultaneous binding of multiple domains to specific RNA structures (Micklem et al., 2000; Ramos et al., 2000). A recent genome-wide study of Staufen-associated RNAs in the early *Drosophila* embryo revealed that the bound RNAs are highly enriched for structures, termed “Staufen recognition structures” (SRS), that are characterised by 19nt long dsRNA regions with no more than 4 mismatches and 4 unpaired bases (19,5,4) that also contain a sub-region with 10/12 paired bases (12,10,2, Laver et al., 2013). We therefore used the RNAfold and RNAplfold algorithms of the ViennaRNA 2.0 package to predict the secondary structure of the *cora* 3׳UTR (Hofacker et al., 2004; Bernhart et al., 2006; Lorenz et al., 2011). This revealed that the RNA is likely to fold into a extensively base-paired structure that contains one strong SRS, in which 19/20 bases are paired, with one bulged nucleotide, and two weaker SRSs with 19/22 base pairs and 17/21 base pairs, the second of which overlaps the end of the coding region (Fig. 7A, B). Furthermore, phylogenetic footprinting reveals that SRS1 has been highly conserved during *Drosophila* evolution, along with large regions of the *cora* 3׳UTR, whereas other non-coding regions, such as the adjacent intron show no conservation (Clark et al., 2007) (Fig. 7C). Thus, the predicted structure of the *cora* 3׳UTR suggests that Staufen is likely to bind to it directly in vivo to mediate its localisation and local translation.

### The cora and oskar mRNA localisation pathways are similar

The discovery that Staufen is required for the localisation of *cora* mRNA to the NMJ raises the question of whether this process is related to any of the three Staufen-dependent mRNA localisation pathways that have already been characterised. There is little to suggest a link with *bicoid* mRNA localisation, as the ESCRTII complex subunit, Vps36-GFP, which binds to the *bicoid 3׳ UTR* and is necessary for the recruitment of Staufen protein (Irion and St Johnston, 2007), does not localise to the NMJ (data not shown). Furthermore, *exuperantia* mutant combinations that disrupt all steps in *bicoid* mRNA localisation have little effect on the levels of GluRIIA at the NMJ, although the morphology of the NMJ is altered (Figure S2A). The basal localisation of Staufen/*prospero* mRNA complexes during the asymmetric divisions of the neuroblasts depends on the binding of Miranda protein to Staufen dsRBD5 (Fuerstenberg et al., 1998; Matsuzaki et al., 1998; Schuldt et al., 1998; Shen et al., 1998). This mechanism of localisation is also unlikely to occur at the NMJ because Staufen dsRBD5 is not required for the normal targeting of *cora* mRNA, although it is necessary for its translation. Furthermore, Miranda protein is not detectably expressed in the muscles (data not shown). However, ectopically expressed Miranda-GFP localises in a rim around the boutons, suggesting that it binds to Staufen that is localised in this region (Figure S2B).

**Fig. 8:**
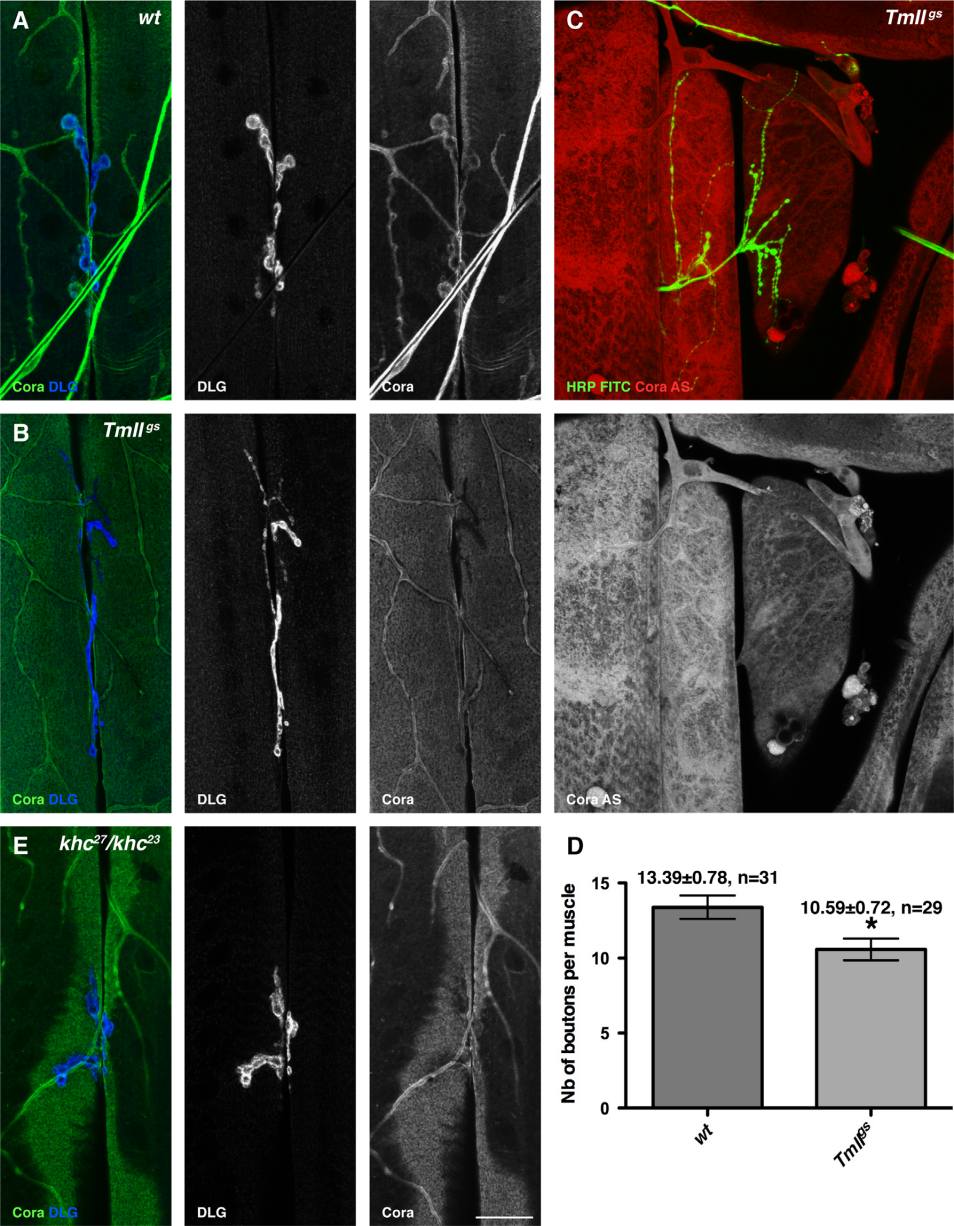
*TmII*^*gs*^ and Kinesin are required for Coracle localisation at the NMJ. (A) A wild type muscle 6/7 NMJ stained for Coracle (green) and DLG (blue). (B) *TmII*^*gs*^ homozygote stained as in (A). Coracle is not localised around the NMJ. (C) ISH to *cora* mRNA in a *TmII*^*gs*^ homozygote. *cora* mRNA (red) is not localised around the NMJ labelled with FITC anti-HRP (green). (D) Quantification of Ib bouton number in muscle 6/7 NMJs in wild-type and *TmII*^*gs*^. *TmII*^*gs*^ mutant NMJs have reduced numbers of boutons (⁎ indicates *p*<0.05). (E) Muscle 6/7 NMJ stained for Coracle (green) and DLG (blue) in a *Khc*^*23*^*/Khc*^*27*^ larva. This combination of kinesin heavy chain alleles disrupts the localisation of Coracle around the NMJs. Scale bar (A: 49 µm; B, E: 25 µ; C: 37 µ). See also Figure S2.

Finally, we tested whether other components of the *oskar* mRNA localisation pathway play a role at the NMJ. The localisation of *oskar* mRNA requires the deposition of the Exon Junction Complex (EJC) on the RNA during the splicing of its first intron and also depends on tropomyosin II (Newmark and Boswell, 1994; Erdélyi et al., 1995; Hachet and Ephrussi, 2001; van Eeden et al., 2001; Hachet and Ephrussi, 2004; Palacios et al., 2004). A null mutant of the EJC component, Barentsz, *btz*^*2*^, had no effect on the localisation of Coracle to the NMJ, consistent with our observation that the unspliced 3׳UTR of *cora* is sufficient to target it to the NMJ (Figure S2C). By contrast, the viable tropomyosin II mutant, *TmII*^gs^, strongly reduced the postsynaptic rim of Coracle around the boutons and reduced the levels of GluRIIA at the NMJ (Fig. 8A and B). Furthermore, *cora* mRNA was not detectably localised at the NMJs of *TmII*^gs^ homozygotes (Fig. 8C). Consistent with this, *TmII*^gs^ mutants had a reduced number of type Ib boutons at the NMJ (Fig. 8D, *wt*=13.39±0.78, *n*=31; *TmII*^*gs*^=10.59±0.72, *n*=29). Thus, the loss of Tropomyosin II and Staufen give very similar phenotypes, suggesting that they function in the same pathway to localise *coracle* mRNA to the NMJ.

*oskar* mRNA is transported along microtubules to the posterior of the oocyte by the plus end directed microtubule motor protein, kinesin, raising the question of whether it transports *cora* mRNA to the NMJ (Brendza et al., 2000). It is not possible to examine *cora* mRNA in *kinesin heavy chain* (*Khc*) null mutants, as these block anterograde axonal transport, leading to progressive paralysis and lethality in the second larval instar (Hurd and Saxton, 1996). We therefore used a hypomorphic combination of Kinesin heavy chain alleles, *Khc*^23^/*Khc*^27^, which develops until the third larval instar (Brendza et al., 1999). This mutant combination caused a reduction or loss of Coracle around the boutons of most NMJs, consistent with a role for kinesin in *cora* mRNA transport (Fig. 8E).

## Discussion

The strength of synapses can be modulated by changing the abundance of neurotransmitter receptors in the postsynaptic membrane, and there is increasing evidence in mammals that the local translation of localised mRNAs can contribute to the modifications taking place in this context. For example, the increase in glutamate receptor levels during synaptic scaling is independent of transcription and requires the local translation of GluR1 mRNA, thereby increasing the ratio of GluR1 to GluR2 (Aoto et al., 2008; Maghsoodi et al., 2008). Similarly, the increase in NMDA receptor levels in hippocampal neurons during long term potentiation involves the local translation of GluN2A, but not GluN2B (Swanger et al., 2013). Our results suggest that the Staufen-dependent regulation of postsynaptically localised mRNAs can regulate the abundance of synaptic glutamate receptors. In this case, however, regulation does not occur through the translational control of the receptors themselves, as GluRIIA protein levels are unchanged in *stau* mutants and *glurIIA* mRNA does not localise to NMJ, but is found throughout the muscle cytoplasm. Furthermore, even if we cannot rule out the existence of an unconventional satellite secretory apparatus beneath the NMJ, the lack of detectable endoplasmic reticulum and Golgi structures at the NMJ makes it unlikely that proteins targeted for secretion are locally translated there.

Instead, we present several lines of evidence to show that Staufen regulates the localisation and local translation at the NMJ of the cytoplasmic scaffolding protein, Coracle. First, we used in situ hybridisation, RNA injection and in vivo RNA tagging to demonstrate that *cora* mRNA localises around boutons. Second, we observed that a *staufen* null mutant disrupts the localisation of both Coracle protein and mRNA. Third, the expression of a Coracle at the NMJ is strongly reduced in the *stau*^*HL*^ mutant, even though *cora* mRNA localisation is unaffected, suggesting that Coracle is locally translated at the NMJ in a Staufen-dependent manner.

Although Staufen may target several mRNAs to the postsynaptic side of the NMJ, it seems likely that the reduced number of synaptic boutons and the decrease in the postsynaptic localisation of GluRIIA in *stau* mutants is due at least in part to this disruption of *cora* mRNA localisation and translation. *coracle* mutant embryos show a dramatic reduction in the levels of GluRIIA at the NMJ but have little or no effect on GluRIIB (Chen et al., 2005). Furthermore, Coracle binds directly to the C-terminal domain of GluRIIA that targets it to the NMJ, leading to the proposal that Coracle anchors the receptor to the actin cytoskeleton (Chen et al., 2005). *staufen* mutant larval muscles show both a strong reduction in Coracle staining around boutons and much lower levels of synaptic GluRIIA, whereas Coracle localisation is normal in *gluRIIA* null mutants. It therefore seems likely that the reduction in the levels of GluRIIA localisation at the larval NMJ are caused at least in part by the effects of *staufen* mutants on Coracle localisation.

While our observations are consistent with a role for Coracle in GluRIIA localisation at later stages, the much larger size of the NMJ in the third larval instar compared to the embryo reveals that the two proteins do not co-localise: GluRIIA is found in clusters in the centre of the bouton opposite the active zones, whereas Coracle lines the periphery of the bouton, just outside the ring of Dlg. This is not compatible with a direct role for Coracle in GluRIIA clustering. Indeed, this role is most probably mediated by Neto, which is also required for GluRIIA localisation, and unlike Coracle, co-immunoprecipitates with the receptor and co-localises with it in clusters in the centre of the bouton (Kim et al., 2012). Instead, we propose that Coracle functions in the delivery of GluRIIA to the synapse. In support of this view, the mammalian Coracle orthologue, 4.1N, regulates the activity-dependent insertion of GluR1 receptors through direct binding to the membrane proximal region of the receptor (Lin et al., 2009). This suggested role of Coracle raises an interesting parallel with the function of Dlg at the NMJ. Dlg is specifically required for the recruitment of GluRIIB to the boutons in the embryo and has no effect on GluRIIA localisation (Chen and Featherstone, 2005). Furthermore, like Coracle, Dlg forms a ring around the periphery of the bouton, with little overlap with the glutamate receptor clusters. Thus, Coracle and Dlg may function in a similar way to regulate the abundance of the two receptor subtypes at the synapse.

### The mechanism of mRNA localisation to the NMJ

Mammalian Staufens have been implicated in mRNA localisation to post-synaptic regions in dendrites and have been found to regulate the stability of a number of mRNAs in neuronal processes (Doyle and Kiebler, 2011; Heraud-Farlow et al., 2013). However, it remains to be proven that Staufen plays a direct role in mRNA localisation to mammalian synapses. Our results demonstrate that the localisation of *coracle* mRNA depends on Staufen protein, providing a functional link between the RNA-binding protein and the postsynaptic localisation of a specific mRNA. Furthermore, our results show that *cora* mRNA is not targeted to the NMJ by a co-translational mechanism, in which the RNA is localised by the binding of the nascent polypeptide to a localised anchor, since the *cora* 3׳UTR is sufficient to mediate localisation in the absence of a coding region. Staufen participates in several different mRNA localisation pathways in *Drosophila* that depend on either microtubules or actin, and *coracle* mRNA could be targeted to the NMJ by a number of possible mechanisms. However, several features of *cora* mRNA localisation resemble the pathway that delivers *oskar* mRNA to the posterior of the oocyte.

First, the C-terminal dsRBD5 of Staufen, which is absent in the *staufen*^*HL*^ mutant as a result of a frameshift, is not required for the targeting of *cora* mRNA to the NMJ. This domain is essential for the microtubule-dependent localisation of *bicoid* mRNA to the anterior of the oocyte and for the actin-dependent localisation of *prospero* mRNA in embryonic neuroblasts (Fuerstenberg et al., 1998; Schuldt et al., 1998; Micklem et al., 2000). *oskar* mRNA localises normally in *stau*^*HL*^ oocytes, as well as in *staufen* null oocytes expressing a form of Staufen lacking dsRBD5 (Micklem et al., 2000). Despite the normal localisation of *oskar* mRNA at the posterior pole of the oocytes, no Oskar protein is produced, indicating that the translation of *oskar* mRNA requires Staufen dsRBD5. It is striking that *stau*^*HL*^ has an identical effect in the muscle, since *cora* mRNA is still localised to the NMJ but no Coracle protein accumulates there. This suggests that Staufen has a second function in the activation of *cora* mRNA translation at the NMJ.

Second, the localisation of *cora* mRNA is strongly reduced in *Tropomyosin II*^*gs*^ homozygotes. Although Tropomyosin II is well known for its essential role as an actin-binding protein, the *TmII*^*gs*^ allele is viable and has no obvious effects on F-actin organisation in the oocyte. Instead, the only known phenotype of this allele is to strongly reduce the posterior localisation of *oskar* mRNA (Erdélyi et al., 1995; Zimyanin et al., 2008). The discovery that *TmII*^*gs*^ also disrupts *cora* mRNA localisation provides another link between the localisations of *osk* and *cora* mRNAs, and highlights the specific effect of this mutation on mRNA transport.

Third, the localisation of Coracle around the boutons is impaired in muscles where kinesin levels have been reduced. The microtubules in the larval muscles radiate from the nuclei and form a basket around the outside of the boutons, close to the area where *coracle* mRNA and protein localise (Ruiz-Canada et al., 2004; Liebl et al., 2005). This organisation is similar to that observed in mammalian muscles, where microtubule plus ends and plus end binding proteins are enriched on the postsynaptic side of the NMJ (Schmidt et al., 2012). Thus, the arrangement of microtubules is consistent with a model in which kinesin transports Staufen/*cora* mRNA complexes to the NMJ. This is an attractive idea, given that mammalian Staufen associates with conventional kinesin in neurons and is a component of RNP particles that undergo kinesin-dependent movements in dendrites (Köhrmann et al., 1999; Kanai et al., 2004). Conclusive proof for a direct role for kinesin in *cora* mRNA transport will require more sophisticated approaches, however, such as imaging of *cora* mRNA motility in *Khc* mutants.

It has recently been reported that some synaptic RNAs, such as *par-6* mRNA, move to the *Drosophila* neuromuscular junction in large RNP particles that bud through the nuclear membrane (Speese et al., 2012). Staufen is not enriched in the nucleus and is therefore unlikely to be a component of these large RNP particles when they assemble in the nucleus, but it would be interesting to determine whether it plays any role in the movement of these particles through the cytoplasm to the NMJ and whether the mechanisms of *cora* and *par-6* mRNAs transport are related given their similar localisations around the synaptic boutons. Our observations also raise the question of whether the cell-biological mechanisms that underlie Staufen-dependent mRNA transport have been conserved between *Drosophila* and mammals and whether dendritic mRNA targeting by mammalian Staufen orthologues requires similar co-factors to *cora* RNA.

## Experimental procedures

### Genetics

#### Alleles

Sequencing of genomic DNA revealed that *stau*^*HL*^ (Schupbach and Wieschaus, 1986; Berg and Spradling, 1991) carries a T to A mutation in the last nucleotide in the intron within the RBD5. The failure to splice this small intron is predicted to produce a protein that lacks dsRBD5, which is replaced by coding sequence from the intron followed by a premature stop codon. Other strains used are as follows: *stau*^*r9*^, (Berg and Spradling, 1991), *Df(2R)Pcl7B* (Duncan, 1982), *dgluR-IIA*^*g9*^ and *Df(2L)clh4* (Petersen et al., 1997), *TmII*^*gs*^ (Erdélyi et al., 1995), *Khc*^*23*^ and *Khc*^*27*^ (Brendza et al., 1999), UAS-DlgA-eGFP (Koh et al., 1999), *UAS-Cora-RNAi* 9788 (VDRC; Vienna, Austria), *24B-Gal4* (P(GawB)how^24B^ (Brand and Perrimon, 1993), *BG57-Gal4* (Budnik, 1996), *M12-Gal4* (P(GAL4)^5053A^ (Ritzenthaler et al., 2000), Ubi-nls-MS2-GFP (Nick Lowe). Wild type strains were either *w*^*1118*^ or *Oregon R*.

### Reporters

UAS-Cora-MS2bs: 10 MS2 binding sites (MS2bs) were cloned into a pBluescript SK vector with an extended polylinker (pUI-MS2bs, Uwe Irion). The 3׳UTR of Coracle was amplified by PCR from Cora EST RE40241 (BDGP) and cloned into pUI-MS2bs. The 3׳UTR-MS2bs fragment was subsequently excised and cloned between SpeI and XbaI in pUASpL.

UAS-Cora-Timer: the DsRed1-E5 fragment was excised from the pTimer-1 vector (Clontech) using BamHI and NotI restriction enzymes, and subcloned into pUASp-PL (pUAS-Timer). Cora-RA 5׳UTR flanked by EcoRI and BamHI restriction sites and Cora-RA 3׳UTR flanked by NotI and SacII restriction sites were subcloned upstream and downstream of the Timer sequence in pUAS-Timer. Both constructs were injected in *w f* embryos.

### Western blots

Body wall muscle extracts were prepared as described previously (Saitoe et al., 1997), run on a 3–8% gradient NuPAGE gels (Invitrogen), transferred to nitrocellulose membranes (Bio-Rad) and sequentially probed with anti-GluRIIA (1:500, Saitoe et al., 1997) and anti-alpha-Tubulin (1:2000, DM1A, Sigma) followed by the corresponding Horseradish Peroxidase (HRP) conjugated secondary antibodies (1:5000, GE Healthcare) and visualised using the ECL Plus Western Blotting detection system (GE Healthcare).

### Immunocytochemistry and immuno electron microscopy

#### Antibodies

Rabbit anti-Staufen (1:100, St Johnston et al., 1991), GluRIIA DM2 GluRIIA (1:500, Saitoe et al., 1997); GluRIIB and GluRIII (1:500, Marrus et al., 2004), guinea pig anti-Cora (1:500, Fehon et al., 1994); anti-DsRed (1:500, Clontech), Mouse anti-Dlg 4F3, GluRIIA 8B4D2 (1:100, Developmental Studies Hybridoma Bank), chicken anti-GFP (1:500, Abcam); anti-rabbit FITC and TxR, anti-mouse Cy5 and TxR (1:250 Jackson Immunoresearch), anti-rabbit Alexa 488, anti-chicken Alexa 488, anti-guinea pig (1:500, Molecular Probes), Cy3 and FITC anti-HRP (1:250 Jackson Immunoresearch Laboratories). In insects, anti-HRP antibodies label axonal membranes (Jan and Jan, 1982).

#### Immunostaining

Third instar wandering larvae were filleted in PBS or Ringer׳s Solution and fixed in PFA 4% in PBS 20 min (for Staufen, GluRIIA DM2, Coracle, DsRed, GFP) or Bouin׳s 5 min (Sigma, for GluRIIA and IIB) or PFA 4% Glutaraldehyde 0.5% in Hepes 0.2 M MgCl2 2 mM pH 7.25 45 min (Immuno EM Staufen). Immunostainings were performed as described previously (Bellen, 2000)

#### EM

Staufen antibody was detected using an HRP coupled anti-rabbit antibody (1:200, GE Healthcare). DAB oxidation (Vectastain, Vector) was carried out in 7.5% sucrose in Tris HCl 0.05 M pH 7.5 to limit diffusion. Fillets were osmicated in 1% OsO4 and flat embedded in Araldite (Fluka). Ultrathin sections (70 nm) of the region between muscle 6/7 were counterstained with Reynold׳s Lead Citrate and aqueous 2% Uranyl Acetate.

### in situ hybridisation

Riboprobes were transcribed from linearised plasmids using the Megascript kit (Ambion) in the presence of Digoxygenin-UTP (Roche) as follows: Cora EST RE40241 (BDGP): antisense NotI/T3, sense XhoI/T7; CG3570 EST GH09390: antisense EcoRI/Sp6; GluRIIA full-length cDNA (gift from Stephan Sigrist) antisense XhoI/T3. Probes were purified on Megaclear columns (Ambion). Fillets dissected in Ringer׳s solution were permeabilised in PBS Tween 0.1%, pre-hybridised for 2 h at 55 °C and hybridised overnight in the presence of 4 µg of probe in standard hybridisation buffer (Hyb). Stringent washes were carried out for 5 h at 55 °C in Hyb. The samples were then stained with Alkaline Phosphatase (AP) coupled anti-Dig (1:200, Roche) and FITC coupled anti-HRP (1:250, Jackson Immunoresearch Laboratories) antibodies overnight. Fast Red solution (Roche) was used as an AP substrate for 1 h at room temperature.

### Injections

The 3׳UTR of Coracle-RA was amplified by PCR and cloned between SacII and EcoRI sites in pUI. After EcoRI linearisation, capped sense RNA (4:1 Cap to GTP ratio, Amersham) labelled with Cy3-UTP (1:10 Cy3-UTP to UTP ratio, Perkin Elmer) was synthesised and purified using the T3 Megascript and Megaclear kits (Ambion). Injections were performed on a customised set up comprising an air table (Thor Labs) a PatchStar injector (Scientifica) on a Nikon Eclipse E800 upright microscope. Dlg-GFP larvae were filleted in HL3.1 buffer (Feng et al., 2004) and individual muscles were injected with a mixture of RNA and food dye to visually monitor the injection (Fast Green FCF, Sigma). After 2 h at room temperature, the fillets were fixed for 20 min in PFA 4%, mounted in vectashield (Vector) and imaged.

### Confocal imaging and quantifications

#### Acquisition

Confocal images are maximum intensity projections of z series acquired with an Olympus FV1000, Zeiss LSM 510 Meta or a Bio-rad 1024. EM was performed using a CM100 Phillips at 120 kV.

#### Bouton numbers

Quantifications were performed on muscle 6 NMJs from segments A2–A4 of 6 larval fillets per genotype. Boutons were quantified from 40x confocal maximum intensity projection of z series from immunostained fluorescent preparations of NMJs stained with anti-HRP and anti-Dlg antibodies. For quantification purposes we defined a bouton as a swelling between neighbouring axonal stretches immunopositive for both anti-HRP and Dlg antibodies. Type Ib boutons were identified by size, and data was compiled using Volocity software (Perkin Elmers). Statistical significance was determined pair-wise using a two-tailed Student׳s *t* test (Prism, Graphpad). All averages are shown with standard errors (SEM).

#### GluRIIA staining intensity

Maximum intensity z projections of muscle 6/7 NMJs simultaneously labelled by anti-Dlg and anti-GluRIIA were used to quantify the intensity of GluRIIA staining using Volocity software (PerkinElmer). We outlined the surface area of the NMJ using the wand tool in the red channel (Dlg) and measured the intensity of the green signal (GluRIIA) in this area (synaptic signal). We also measured the intensity of the green signal outside the NMJ for each muscle (extrasynaptic signal). The average synaptic signal obtained for *gluRIIA* null mutants was used to measure the background noise. This figure was subtracted from each average. We then calculated the synaptic ratio by dividing the synaptic signal by the extrasynaptic. The resulting values were normalised.

### RNA secondary structure prediction

The secondary structure of the *cora* 3׳UTR was predicted using the RNAfold and RNAplfold algorithms of the ViennaRNA 2.0 package (Hofacker et al., 2004; Bernhart et al., 2006; Lorenz et al., 2011).

## Appendix A Supplementary materials

Supplementary data: Fig. S1 The secretory machinery is not localised to the subsynaptic region of the *Drosophila* NMJ. (A) A wild type muscle stained with anti-HDEL (green) and synaptotagmin (red). The endoplasmic reticulum, stained with an anti-HDEL antibody, is localised around the nuclei, and is not detectable beneath or near the boutons of the NMJ, marked by synaptotagmin (red). (B) A wild type muscle stained for the trans-Golgi marker, Golgin 245 (green) and DLG (red). The Golgi ministacks are uniformly distributed throughout the cytoplasm with no obvious enrichment around the NMJ. (C) A wild type muscle stained for the cis-Golgi marker, GM130 (green) and DLG (red). (D) Double-labelling of GM130 (cis-Golgi, green) and dGCC88 (trans-Golgi, red) to show the close juxtapositioning of the cis and trans-Golgi, which do not cluster around DLG positive boutons (blue). Scale bar (A: 25 µm; B–D: 15 µm).

Supplementary data: Fig. S2 GluRIIA localisation appears normal in *barentsz* and *exuperantia* mutants. (A) NMJ between muscles 6/7 from an *exu*^QR^/*Df* larva stained for DLG (red) and GluRIIA (green). (B) Ectopically expressed Miranda-GFP localises around the NMJ labelled with Cy3 anti-HRP. (C) NMJ between muscles 6/7 from a *btz*^2^ larva stained for DLG (red) and Coracle (green). Scale bar (A: 25 µm, B: 24 µm, C: 37 µm).
