# Corrigendum to “Staufen targets *coracle* mRNA to *Drosophila* neuromuscular junctions and regulates GluRII asynaptic accumulation and bouton number” [Dev. Biol. 392 (2014) 153–167]

**DOI:** 10.1016/j.ydbio.2014.07.012

**Published:** 2014-09-15

**Authors:** Alejandra Gardiol, Daniel St Johnston

**Affiliations:** The Wellcome CRUK Gurdon Institute, University of Cambridge, Tennis Court Road, Cambridge CB2 1QN, United Kingdom

It has come to the attention of the authors that Bélanger et al. (Bélanger, G., Stocksley, M.A., Vandromme, M., Schaeffer, L., Furic, L., Desgroseillers, L., Jasmin, B.J., 2003. Localization of the RNA-binding proteins Staufen1 and Staufen2 at the mammalian neuromuscular junction. J. Neurochem. 86, 669–677) reported that Staufen 1 and 2 localise to the postsynaptic side of mouse neuromuscular junctions, suggesting that the function of Staufen at the NMJ is conserved in mammals.

