# Supplementary figures and images for: Staufen targets coracle mRNA to Drosophila neuromuscular junctions and regulates GluRIIA synaptic accumulation and bouton number
Source: Dev Biol. 2014 Aug 15;392(2):153–67. doi: 10.1016/j.ydbio.2014.06.007 (PMC4111903; doi:10.1016/j.ydbio.2014.06.007)

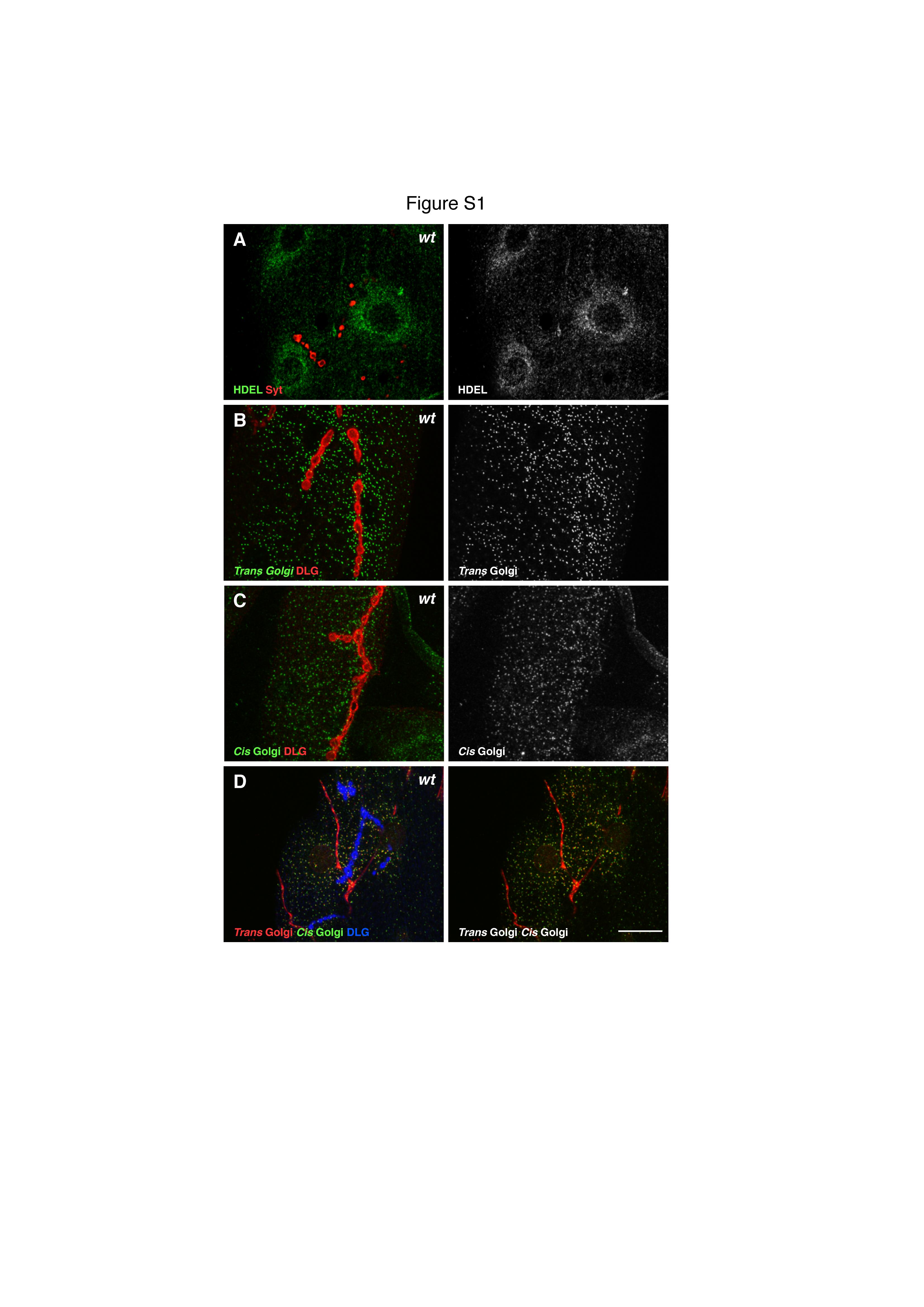

Supplement: Supplementary file 1 — Supplementary data: Fig. S1 The secretory machinery is not localised to the subsynaptic region of the Drosophila NMJ. (A) A wild type muscle stained with anti-HDEL (green) and synaptotagmin (red). The endoplasmic reticulum, stained with an anti-HDEL antibody, is localised around the nuclei, and is not detectable beneath or near the boutons of the NMJ, marked by synaptotagmin (red). (B) A wild type muscle stained for the trans-Golgi marker, Golgin 245 (green) and DLG (red). The Golgi ministacks are uniformly distributed throughout the cytoplasm with no obvious enrichment around the NMJ. (C) A wild type muscle stained for the cis-Golgi marker, GM130 (green) and DLG (red). (D) Double-labelling of GM130 (cis-Golgi, green) and dGCC88 (trans-Golgi, red) to show the close juxtapositioning of the cis and trans-Golgi, which do not cluster around DLG positive boutons (blue). Scale bar (A: 25 µm; B–D: 15 µm). [file mmc1.zip › mmc1.tif]

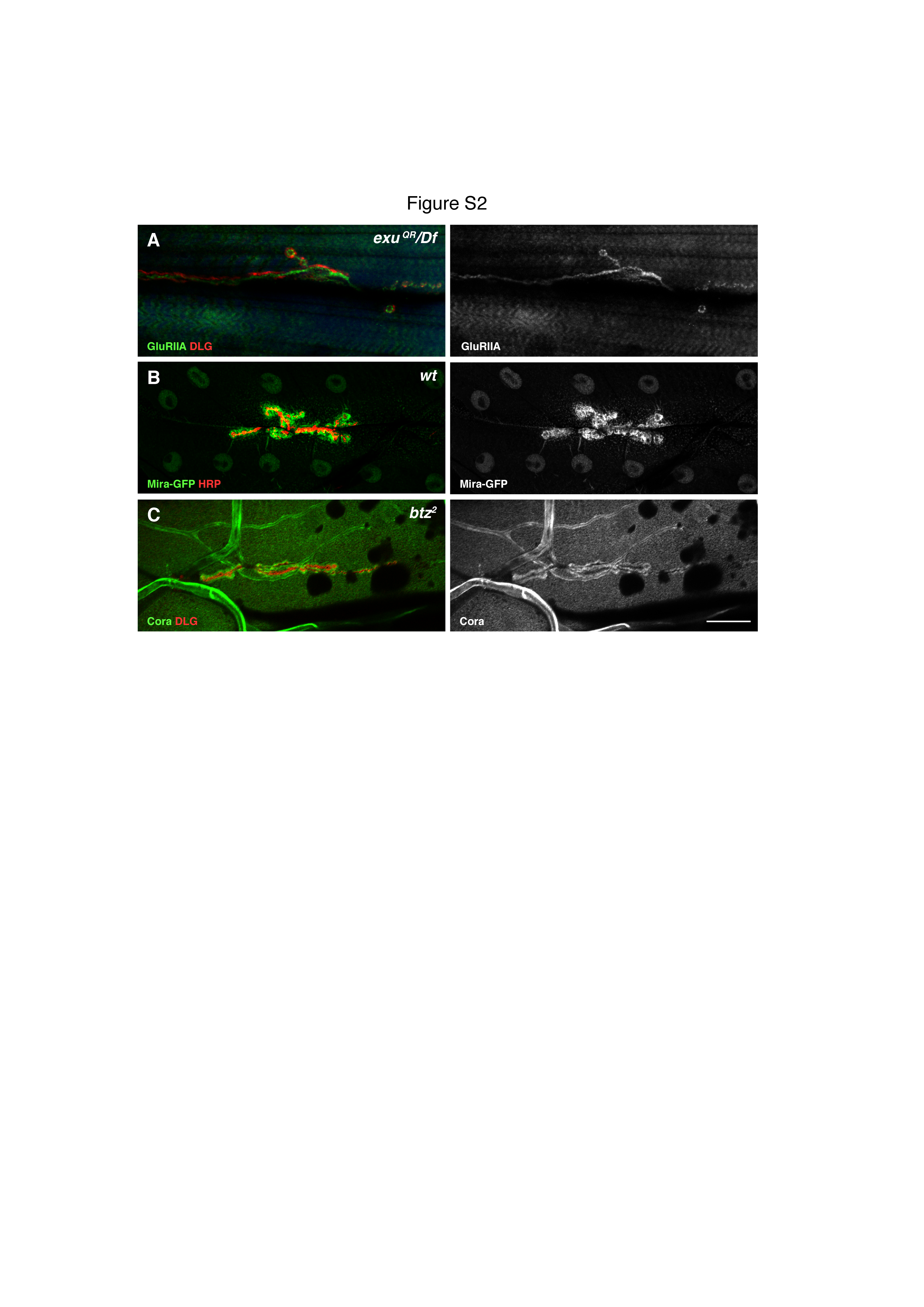

Supplement: Supplementary file 2 — Supplementary data: Fig. S2 GluRIIA localisation appears normal in barentsz and exuperantia mutants. (A) NMJ between muscles 6/7 from an exuQR/Df larva stained for DLG (red) and GluRIIA (green). (B) Ectopically expressed Miranda-GFP localises around the NMJ labelled with Cy3 anti-HRP. (C) NMJ between muscles 6/7 from a btz2 larva stained for DLG (red) and Coracle (green). Scale bar (A: 25 µm, B: 24 µm, C: 37 µm). [file mmc2.zip › mmc2.tif]
